# Quantifying Lifespan Cognitive Health Risk: Findings from a longitudinal twin study

**DOI:** 10.1002/alz70860_105064

**Published:** 2025-12-23

**Authors:** Sophie A. Bell, Christopher R. Beam, Alyssa C Kam, Ebrahim Zandi, Deborah Winders Davis, Deborah Finkel, Eric Turkheimer

**Affiliations:** ^1^ University of Virginia, Charlottesville, VA, USA; ^2^ University of Southern California, Los Angeles, CA, USA; ^3^ University of Louisville School of Medicine, Louisville, KY, USA; ^4^ Norton Children's Research Institute affiliated with the University of Louisville School of Medicine, Louisville, KY, USA; ^5^ Jönköping University, Jönköping, Sweden

## Abstract

**Background:**

Effective prevention and intervention for Alzheimer's disease and related dementias (ADRD) depend on understanding the mechanisms driving pathology decades before clinical symptoms emerge. Developing tools that quantify lifelong risk accumulation is crucial for identifying asymptomatic individuals on a trajectory toward cognitive decline. The Louisville Twin Study (LTS) is the first twin study to combine prospective childhood developmental data with comprehensive midlife assessments to investigate risk and protective factors for cognitive decline.

**Method:**

We present results from 432 middle‐aged twins (89 monozygotic pairs, 66 dizygotic pairs). All IQ scores were based on in‐person WISC and WAIS‐IV assessments. Average age for childhood IQ assessment was 13.8 ± 2.4 years, and average age for midlife follow‐up was 47.6 ± 10.4 years. Childhood IQ was the strongest predictor of adult IQ in our study (*r* = 0.79). By including childhood IQ as a predictor in our regression models, we identified risk and protective factors associated with change in IQ between childhood and midlife. We then computed Lifespan Cognitive Health Risk (LCHR) scores using two methods: a regression‐weighted formative latent variable and a unit‐weighted sum of all predictors to minimize in‐sample bias. These results were followed up with genetically informed twin analyses that identified phenotypic associations between LCHR and cognitive decline, independent of genetic and shared environmental confounds.

**Result:**

Higher childhood socioeconomic status and educational attainment were protective against cognitive decline, while childhood trauma, midlife smoking, psychopathology severity, lung function, gait speed, grip strength, and difficulty completing instrumental activities of daily living were risk factors. Genome‐wide data from the LTS allowed us to identify a polygenic index for cognitive ability and accelerated DNA methylation age as risk factors. Within identical twin pairs raised together, the twin with a higher LCHR score had a greater decline in IQ between childhood and midlife.

**Conclusion:**

The LCHR is a multidomain risk index composed of feasible, inexpensive measures that can be collected in typical clinical or research settings. This risk index builds on existing prediction models for ADRD and cognitive decline by leveraging a “quasi‐causal” twin framework, which strengthens causal inference between risk and protective factors and cognitive decline.

